# Device-Free Passive Identity Identification via WiFi Signals

**DOI:** 10.3390/s17112520

**Published:** 2017-11-02

**Authors:** Jiguang Lv, Wu Yang, Dapeng Man

**Affiliations:** Information Security Research Center, Harbin Engineering University, Harbin 150001, China; lvjiguang@hrbeu.edu.cn (J.L.); mandapeng@hrbeu.edu.cn (D.M.)

**Keywords:** WiFi, channel state information, human gait, human identification

## Abstract

Device-free passive identity identification attracts much attention in recent years, and it is a representative application in sensorless sensing. It can be used in many applications such as intrusion detection and smart building. Previous studies show the sensing potential of WiFi signals in a device-free passive manner. It is confirmed that human’s gait is unique from each other similar to fingerprint and iris. However, the identification accuracy of existing approaches is not satisfactory in practice. In this paper, we present Wii, a device-free WiFi-based Identity Identification approach utilizing human’s gait based on Channel State Information (CSI) of WiFi signals. Principle Component Analysis (PCA) and low pass filter are applied to remove the noises in the signals. We then extract several entities’ gait features from both time and frequency domain, and select the most effective features according to information gain. Based on these features, Wii realizes stranger recognition through Gaussian Mixture Model (GMM) and identity identification through a Support Vector Machine (SVM) with Radial Basis Function (RBF) kernel. It is implemented using commercial WiFi devices and evaluated on a dataset with more than 1500 gait instances collected from eight subjects walking in a room. The results indicate that Wii can effectively recognize strangers and can achieves high identification accuracy with low computational cost. As a result, Wii has the potential to work in typical home security systems.

## 1. Introduction

Identity identification has been researched for many years and there have been several methods for human identification such as fingerprint-based [1], face recognition-based [2] and iris-based [3] methods. It is widely used in security systems as these biological characteristics are unique among people and can provide high identification accuracy. The characteristics can be utilized in the security systems to make sure whether someone has access to a certain room. However, most identity identification methods either need wearable devices or participating actively in the identification process, which hinders their applicability in some specific scenarios such as intrusion detection. 

WLAN-based device-free passive sensing attracts much attention in recent years because it does not require the user to be equipped with any sensing devices or behave actively in the sensing process [4]. WiFi has been widely deployed in our daily life that it can provide Internet access for a large number of devices. Numerous researchers find that WiFi signal can be utilized in device-free sensing as WiFi devices can not only be used for communication, but also act as generalized sensors. The movement of human has an impact on signal transmission, and different people’s gait may cause different multipath to signal propagation. It is confirmed that human’s gait is different from each other and it can be seen as his unique characteristic. This motivates our research on WLAN-based device-free identity identification. Compared to traditional security systems, which are based on video cameras or infrared equipment, WLAN-based approaches do not have the disadvantages of privacy leakage [5] and high cost. There have been several pioneering studies on WLAN-based device-free identity identification [6,7,8]. These approaches leverage Channel State Information (CSI) in physical layer of wireless networks to model human’s gait.

However, there are still many challenges in WiFi-based human identification. One of the biggest challenges is how to extract effective gait patterns using CSI dynamics. The received CSI waveform contains the signal reflections of the whole body. As a result, it is difficult to divide the CSI time series into certain segments according to walking steps. In addition, CSI feature extraction is also a challenging task for human identification. In other words, we should find proper features to characterize human’s gait. The common features such as signal variance, median value, and mean value are not effective because they can be easily influenced by environmental changes. Thus, there is a huge gap between the extracted feature and the exact gait character.

To deal with these challenges, in this paper, we present Wii, a device-free WiFi-based Identity Identification approach. It is composed of four main modules, which are pre-processing, step segmentation, feature extraction and classification. Classification contains two sub-modules, stranger recognition and identity identification. First, PCA and low pass filter are utilized to eliminate the useless signal components and high frequency noise. Then, walking steps are automatically segmented by Continuous Wavelet Transform (CWT) and wavelet variance. Thirdly, several time and frequency domain features are extracted before a few of them are selected according to information gain to represent human’s gait. Finally, a Gaussian Mixture Model (GMM) is utilized to recognize strangers and a Support Vector Machine (SVM) with Radial Basis Function (RBF) kernel acts as the classifier to identify people.

We implement a prototype system in a 5 m × 4 m meeting room with eight volunteers for evaluation and compare the performance with WiWho and FreeSense. The results show that the stranger recognition rate can achieve over 91% and the identity identification accuracy can achieve 90.9% to 98.7% when the size of the candidate user set changes from 8 to 2, which is feasible in common indoor scenarios.

In summary, the main contributions of the paper are as follows.

- We propose Wii, a novel device-free WiFi-based identity identification approach, which is capable of recognizing strangers who are outside the training set and has a higher human identification accuracy compared to existing methods.
- We use PCA and low pass filter to remove the useless components in the WiFi signals and reduce the computational complexity at the same time.
- We combine CWT and wavelet variance to segment CSI waveforms into single walking steps. It is based on the observation that the wavelet coefficients show obvious periodicity.
- We extract and select gait features from both time and frequency domain according to information gain that can better represents human’s walking patterns and reduce computational cost. As a result, the identification accuracy becomes higher.

The rest of this paper is organized as follows. Section 2 presents the related work in human identification and device-free sensing. The basic background knowledge of CSI of WiFi network is introduced in Section 3. In Section 4, we present the detail design of Wii, and experimental evaluations are presented in Section 5. Then we discuss the potentials and limitations of Wii in Section 6. Finally, we conclude our work in Section 7.

## 2. Related Work

A broad range of identity identification approaches have been proposed these years that can satisfy different scenarios.

Most identity identification approaches leverage biometric characteristics such as fingerprint, iris and even gait. Iris is widely used in user authentication as it is unique among different people and very stable across different time. An iris recognition system is proposed in [9] including two modules, localizing iris and iris pattern recognition. A digital camera is used to capture images, from which iris is extracted. The iris is then reconstructed into rectangle format and iris pattern is recognized. Park et al. propose an iris recognition approach based on score level fusion, in which two Gabor wavelet filters and SVM are used [3]. Daugman’s Rubber Sheet Model is proposed to extract features from iris including freckles, coronas and stripes [10]. Fingerprint is also widely used to identify a person, as it has high uniqueness. In fingerprint-based user authentication, some special points called minutiae points are extracted from fingerprint images. The ridge thinning algorithm is put forward to extract minutiae features from the fingerprint [11]. 

Although the recognition accuracy of the above techniques is relative high, they all require specific infrastructure, which limits their applicability. Recently, many researchers pay their attention on passive recognition schemes using wireless sensing, especially WLAN-based frameworks. There are many applications in WLAN-based wireless sensing as it does not require the user to carry any devices and it can even only use WiFi infrastructure to achieve high accuracy sensing such as human detection, activity recognition and identity recognition.

Human detection and crowd counting are the fundamental processes of indoor localization and identity recognition. Researchers first implement human detection systems utilizing received signal strength (RSS), as it is easy to obtain from many kinds of wireless devices. Human presence can affect the propagation of wireless signal and hence received signal strength varies as people move [12,13,14,15]. However, RSS is a coarse-grained measurement that it has weak stability due to severe multipath effect in indoor scenario. More recently, researchers find channel state information has better properties in physical layer. As illustrated in [16], CSI is a more fine-grained measurement of wireless signal compared with RSS as it is a subcarrier level measurement and contains both amplitude and phase, and it can be extracted from commodity devices with slight firmware modification. As a result, CSI-based passive human detection approaches attract much attention. Most CSI-based methods treat CSI as extended RSS, such as [17]; the variance of CSI amplitude is extracted as the feature to detect human motion. PADS extracts features from both amplitude and phase of CSI to make human detection system more sensitive and it can detect human of different moving speeds [18]. A proper feature extracted only from amplitude of CSI can also be sensitive enough to detect human even if the moving speed is very slow [19]. Wireless signals can be used to provide a rough estimation of the number of people in the monitoring area. Different numbers of people usually generate different signal measurements due to multipath effect in indoor scenarios [20,21,22,23].

Device-free passive sensing can also be used in activity recognition. E-eyes can recognize a few daily activities such as sleeping and cooking using CSI histograms as features [24]. CARM is a model-based activity recognition system [25]. It models the relationship between CSI and human activities. WiDraw can track the activity of a person’s hand according to the Angle of Arrival (AoA) of WiFi signal [26]. Smokey can detect if a person I s smoking using WiFi signal even under NLOS environment [27]. WiFinger is presented to recognize the minute variation of signal when the finger moves [28].

It is confirmed that gait is a unique feature of a person, as a result of which it can be used to identify a subject [29]. WiFi-ID extracts representative features of people’s walking style and shows for the first time that WiFi signal can be used to identify a person [8]. WiWho is a framework using WiFi signal to identify a person’s step and walking gait [6]. It shows that identity can be identified based on step and walk analysis. WifiU profiles human movement in which spectrograms are generated from CSI [30]. FreeSense combines PCA, Discrete Wavelet Transform (DWT) and Dynamic Time Warping (DTW) techniques to implement human identification [7]. 

Different from the approaches above, we for the first time calculate the periodicity from the CSI of WiFi signal using Continuous Wavelet Transform (CWT), and extract time and frequency domain features from a single step as well as a few steps. As a result, the identification accuracy of Wii can be higher than the above approaches. Furthermore, Wii can recognize strangers from a group of people.

## 3. Preliminary

Wii utilizes CSI in the physical layer of WiFi signal. Consequently, in this section, we give a brief introduction of CSI.

In a common indoor scenario, the wireless signal propagates through multiple paths to the receiver. There may exist LOS path and several reflection paths, and the received signal is the superposition of signals from different paths. In OFDM system, the wireless channel in the time domain can be descripted by a Channel Impulse Response (CIR) to distinguish different paths. Under the assumption of time-invariant, CIR can be expressed as:

(1) $$h(\tau )=\sum_{i=1}^{N}\alpha_{i}e^{-j\theta_{i}}\delta (\tau -\tau_{i})+n(\tau ),$$

where $\alpha_{i}$, $\theta_{i}$, and $\tau_{i}$ denote the amplitude, phase and time delay of the signal from ith path, respectively; N is the total number of paths; n(τ) is complex Gaussian white noise; and δ(τ) is the Dirac delta function.

However, precise CIR cannot be extracted from ordinary commodity infrastructures which is inapplicable in home environment. To overcome this limitation, in the frequency domain, Channel Frequency Response (CFR) can model the transmitting channel, which is composed of amplitude-frequency response and phase-frequency response. Given inﬁnite bandwidth, CIR is equivalent to CFR, and CFR can be derived by taking the Fast Fourier Transform (FFT) of CIR:

(2) H=FFT(h(τ)).

The Linux kernel driver of Intel 5300 NIC can be slightly modified to make it convenient to obtain CFRs for N=30 subcarriers in the format of CSI:

(3) $$H=[H(f_{1}),H(f_{2}),\ldots ,H(f_{N})].$$

The amplitude and phase of a subcarrier can be described by CSI:

(4) $$H(f_{k})=\left\|H(f_{k})\right\|e^{j\sin(∠H)},$$

where $H(f_{k})$ is the CSI of the subcarrier of which the central frequency is $f_{k}$, and ∠H denotes its phase. Therefore a group of CSIs $H(f_{k}),(k=1,\ldots ,K)$, reveals K sampled CFRs at the granularity of subcarrier level.

## 4. System Design

In this section, the overview of Wii is presented followed by the design details of each module.

### 4.1. System Overview

**Figure 1:**
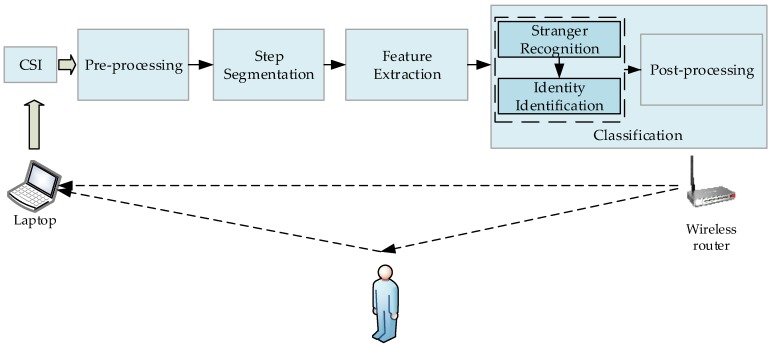
System framework.

The framework of Wii is provided in Figure 1; it has four main components: pre-processing, step segmentation, feature extraction and classification. In the classification procedure, it contains stranger recognition and identity identification before post-processing. Noise removing is conducted in preprocessing as there are more than one kind of noise included in the wireless signal. Step segmentation process can divide continuous CSI data into discrete segments according to walking steps in order to extract per-step features. In feature extraction module, several time and frequency domain features are extracted and the features that generate the largest information gain are selected. Finally, stranger is recognized and identity is identified in classification module. 

The system works in a typical indoor environment with a pair of commodity WiFi devices deployed. A wireless router that supports IEEE 802.11n protocol acts as the TX and a laptop equipped with some certain model of wireless network interface card (such as Atheros 9390 and Intel 5300) acts as the RX as well as the server. They keep transmitting data to collect CSIs when a person walks in the monitoring area. The collected CSIs are stored in the laptop, and will be processed in the subsequent procedures. 

### 4.2. Pre-Processing

During our experiments, we find that the number of collected CSIs is not the same as the number of transmitted ICMP packets we set. Thus, we first conduct the linear interpolation in the raw collected CSIs to calibrate the sampling frequency. As a result, the collected CSIs have a unified sampling frequency that we can then extract frequency domain features after interpolation. According to the theory of Orthogonal Frequency Division Multiplexing (OFDM), the subcarriers of the channel are independent and have different frequency which causes frequency selective fading [31]. However, actually, the CSI data of adjacent subcarriers have some relationships. For each ICMP packet, a CSI matrix of 3 × 30 can be extracted. Consequently, we use PCA to obtain the independent data. PCA can automatically combine the correlated CSI streams to extract more representative components. The CSI matrix is reshaped into a 1 × 90 vector. It consists of a *n* × 90 matrix for *n* collected CSI vectors in a time interval. We calculate the principle components of the matrix and find that, in most cases, the contribution rate of the first principle component is higher than 85%, thus we use the first principle component as the representation of walking data. 

However, there are several kinds of noises reserved in the first principle component that have negative effect on the identification accuracy. The one that has the most significant negative influence is high frequency noise due to other movements. The signal components that reflected by torso, arms and legs are useful in identifying identity. The frequency of these components is lower than 10 Hz according to the intuition and our observation. Consequently, we utilize a low pass filter with the cutoff frequency of 10 Hz to eliminate the high frequency noise in the walking data. After pre-processing, most noise in the raw CSIs has been removed.

### 4.3. Step Segmentation

Our identity identification system is based on human’s gait information, so it is necessary to find the start and end point of each step from the collected data to divide the data sequence into segments according to their walking steps. 

Generally, a person’s walking speed is constant in a short time and therefore, there is an obvious periodicity during the walking period. However, it is challenging using wireless signal to determine the periodicity of walking. Unlike the device-based methods such as inertial sensor-based systems, in which the sensor is attached to human body and it is easy to determine the periodicity. We cannot observe an obvious periodicity directly from the CSI waveform. As a result, we need to dig deep down into different frequency components using time-frequency analysis techniques to find the periodicity pattern. Fortunately, the combination of Continuous Wavelet Transform (CWT) and wavelet variance can effectively discover periodicity in a multi-scale manner from the waveform.

**Figure 2:**
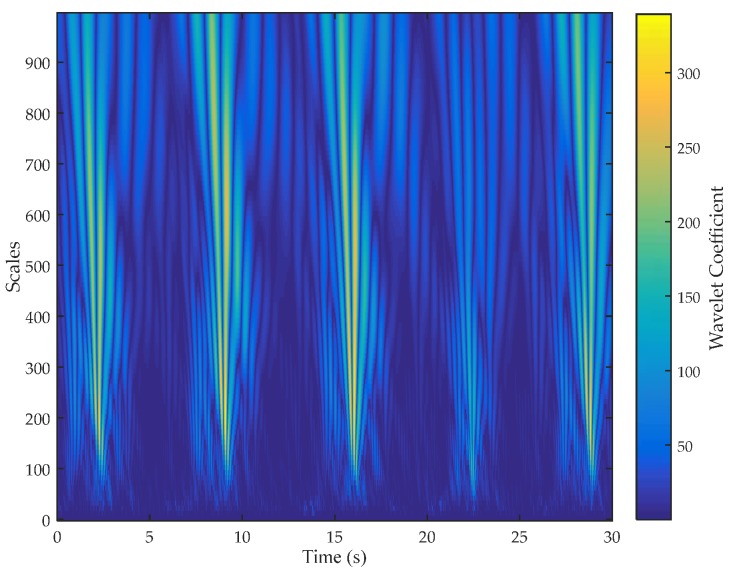
Wavelet coefficient of multiple scales of channel state information.

First, we calculate the wavelet coefficients of the first PCA component of the CSI waveform after low-pass filtering (*cpl*) in multiple scales using CWT. According to our experimental results of trying different wavelet functions, we choose DB6 (Daubechies) wavelet [32]. As shown in Figure 2, we can see clearly the periodicity of the wavelet coefficients exists under some scales. However, the exact periodicity cannot be confirmed intuitively from the wavelet coefficients. The wavelet variance reflects the distribution of the power of the wavelet coefficients in different scales, and it can be used to estimate the main periodicity of a time series [33]. Thus, we then calculate the wavelet variance as Equation (5).

(5) $$\mathrm{var}(a)=\int_{-\infty }^{+\infty }{\left|W_{f}(a,b)\right|}^{2}db,$$

where ${\left|W_{f}(a,b)\right|}^{2}$ is the power of the wavelet coefficient of scale a at time b.

**Figure 3:**
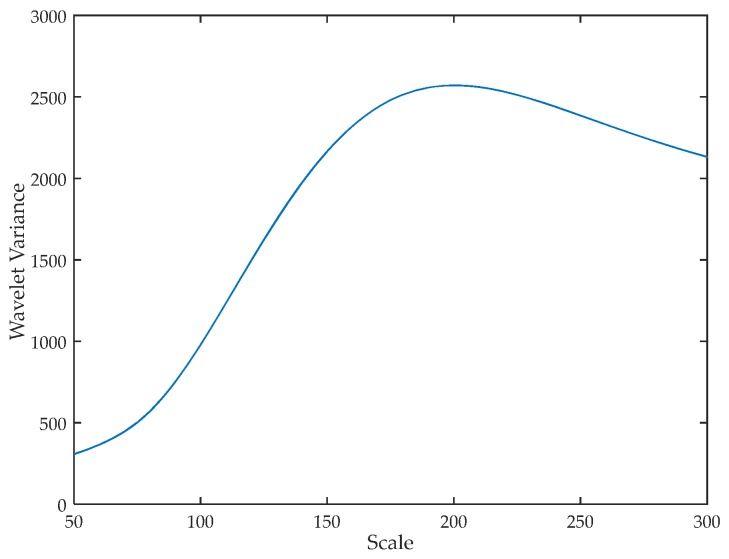
Wavelet variance.

Second, as indicated in Figure 3, we can see that the maximum wavelet variance locates at the scale of about 190, which means the wavelet coefficients have the most obvious periodicity at this scale. Based on the observation of our experiments, this periodicity is mainly caused by the movement of torso, as the torso affects the signal transmission most significantly.

It is noteworthy that the location of the maximum wavelet variance varies among different people and even different time of the same person as the walking speed is not the same among different people and not constant during walking. Fortunately, Wii is a window-based approach, and it can calculate the periodicity dynamically when people walk at the definition of a certain time window. As a result, Wii has the ability to adapt to different walking speeds of a person.

**Figure 4:**
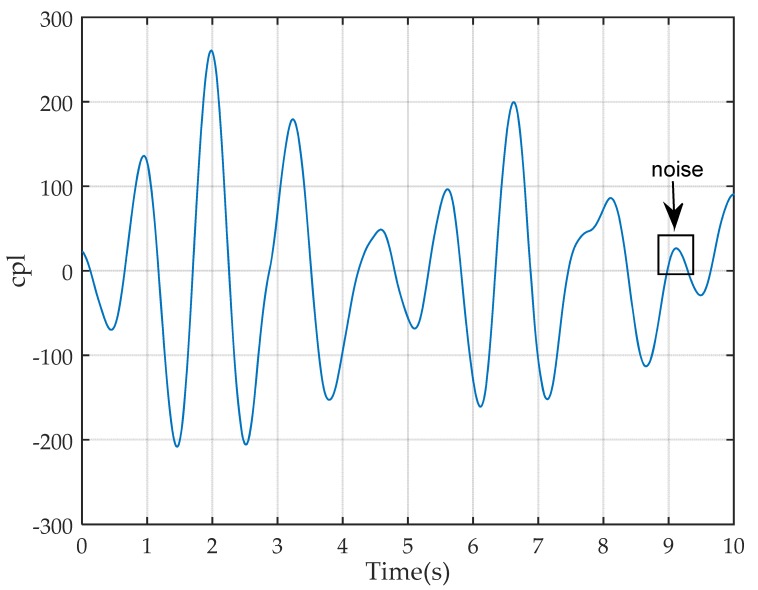
Wavelet coefficient waveform of steps.

Third, we can use this scale to extract the corresponding *cpl* that is relative to walking. When collecting CSI data, we also use a digital camera to take video records of walking. Then, we compare the waveforms of *cpl* of this scale ($cpl^{s}$) with video records and find that the waveform between two zero crossing points can represent one step as shown in Figure 4. However, some noises in the waveform exists; that is, the time interval between two adjacent zero crossing points is too short or too long. Thus, we set the lower bound $t_{lb}$ as 0.5 s and upper bound $t_{ub}$ as 1.2 s intuitively as the time interval of most steps are between these two bounds. 

Finally, the $cpl^{s}$ can be divided into step segments according to the locations of these points with the time interval between $t_{lb}$ and $t_{ub}$.

### 4.4. Feature Extraction

**Figure 5:**
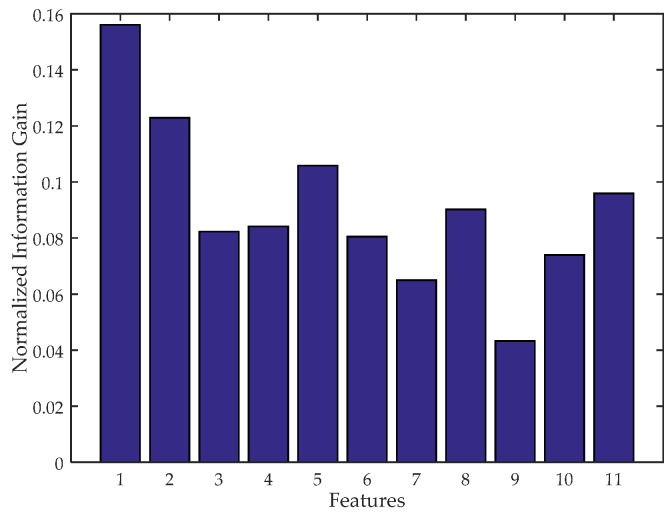
Normalized information gain of the candidate features.

During our experiments, we have extracted many features such as the maximum, minimum, mean and some other statistic features of the waveform, and find that both time and frequency domain features can be used to represent the characteristics of human walking. In addition, per-step features and walking features can be both extracted in identity identification. However, these features should be calculated in a certain window. Consequently, we use two types of windows, step window and walking window. A step window is the time length of one step and a walking window contains several step windows. The candidate features calculated in this paper are listed in Table 1 with Equations (6)–(16). In the equations, *n* is the number of $cpl^{s}$ in a step, *m* is the number of steps in a walking window, $cpl_{i}^{s}$ is *i*th $cpl^{s}$ in the step, and $cpl^{s}{}_{j}$ is the $cpl^{s}$ of *j*th step. In equation (16), we divide $cpl^{s}$ values in a step into 10 segments and $p_{k}$ is the ratio of the number of $cpl^{s}$ of *k*th segment to the total number of $cpl^{s}$ in the walking window. However, too many features will lead to a high computational complexity and even overfitting that the identification accuracy decreases. To select the most effective features, we calculate the normalized information gain of each feature to quantify their impact on classification as shown in Figure 5, and select the Top 5 features [34]. A higher information gain value means a higher capability of representing the characteristic. As a result, we choose the maximum (max), minimum (min) and standard deviation (std) of $cpl^{s}$ as the time-domain features in step windows, average step time (ast) as the time-domain feature in walking windows, and entropy as the frequency-domain feature in step windows. 

In the experiments, we set the walking window length from 2 to 10, while the window step is 1 walking step, and the evaluation results will be illustrated in Section 5.

### 4.5. Training and Classification

As a person’s walking path has a great impact on the features of wireless signal because of multipath effect, usually the identity identification system is placed near the entrance of the room or a corridor that people in the area walk in a relative fixed path. Thus, scenarios of training and classification are the same, which ensures the effectiveness of training samples. 

After extracting the per-step and walking features of human walking, we integrate the features of several steps to build people’s gait profiles. Wii first utilizes Gaussian Mixture Model (GMM) to distinguish between authenticated people and strangers and then uses the multi-class SVM with Radial Basis Function (RBF) kernel as the classifier to identify people based on the extracted features. The inputs of GMM are the vectors of selected feature values of strangers and authenticated people, and the outputs of GMM are probabilities whether the steps belong to a stranger or an authenticated person. The inputs of SVM are the vectors of selected feature values of the authenticated people, and the outputs of SVM are probabilities that each step’s owner.

It is difficult for a classifier to recognize a subject outside the training set. As a result, we collect several samples of strangers and construct a stranger profile. The samples are constructed by the five features with the highest information gain. The authenticated people can be seen as a Gaussian model while strangers can be seen as another Gaussian model. Consequently, we use GMM to recognize strangers to authenticate people in the monitoring area. The strangers used in the training data are only some samples of the assumed strangers. However, when a genuine stranger comes, his similarity is higher to the samples of strangers than the authenticated people. As a result, a genuine stranger can also be recognized. After the authenticated people are recognized, the identity will be identified. In the identification phase, Wii extracts the same features as the training phase and uses the SVM to identify people. We use the LIBSVM toolbox proposed by Chih-Jen Lin [35] which is widely used in machine learning.

In the end of classification, we have a post-processing procedure, which can further improve the identification accuracy. In the post-processing, we assume that the successive steps in a walking window belong to the same person. It is unlikely to happen in real scenarios that Person A suddenly disappears and Person B shows up to walk for a single step or two steps instead of Person A, and Person A appears again to continue walking. For example, if the identification result sequence in a walking window is AABAA, we can replace B in the middle with A in the identification result in this situation. The only cost of post-processing is the delay in presenting the identification result, but the recognition can be more accurate.

## 5. Evaluation

### 5.1. Experiment Setup

**Figure 6:**
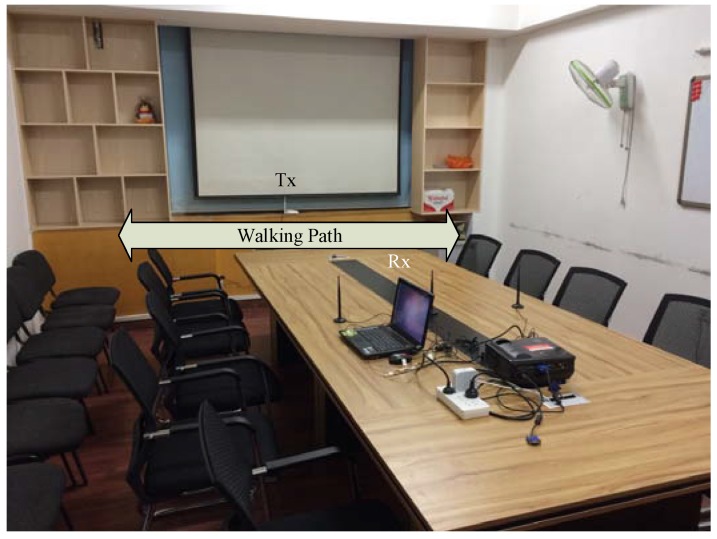
Experimental scenario.

To evaluate the performance of Wii, we conduct real experiments using holdout cross validation in a typical meeting room with the size of 5 m × 4 m as shown in Figure 6. A part of data is randomly selected as the training data, while the other part is used as test data. This procedure is repeated five times in each evaluation. Each result is the mean value of the five validations. The meeting room is occupied with a meeting table and chairs. We use a TP-Link 802.11n wireless router with a single antenna as the transmitter and a Lenovo laptop equipped with a three-antenna Intel WiFi Link 5300 (iwl 5300) NIC running Ubuntu 10.04 OS as the receiver. Specifically, the antennas of the laptop are modified using three 6 dbi gain antennas, and the firmware of the NIC is modified to extract CSIs from data packets using the CSI tools. The transmitter and receiver are placed about 0.75 m above the floor and 3 m away from each other.

During data collection period, the laptop is configured to continuously send ICMP packets to the wireless router and it will receive corresponding response packets. To collect CSI data to obtain precise information of people’s walking activity, the sampling rate should be as high as possible. However, the processing ability of the wireless router is limited, we adjust the sampling rate to be 200 Hz in consequence. The NIC records a CSI sample with CFR of 3 × 30 subcarriers from each respond packet. We recruit eight healthy volunteers in our experiment and the basic information of the volunteers is shown in Table 2. The volunteers are asked to walk naturally on the path that crosses the LOS of the transceivers without the constraints of walking speed or style. The data collection takes five days and a volunteer walks without anyone else in the meeting room to reduce the noise. After step extraction, we get about 150 steps for each volunteer.

### 5.2. Performance Evaluation

#### 5.2.1. Stranger Recognition

**Figure 7:**
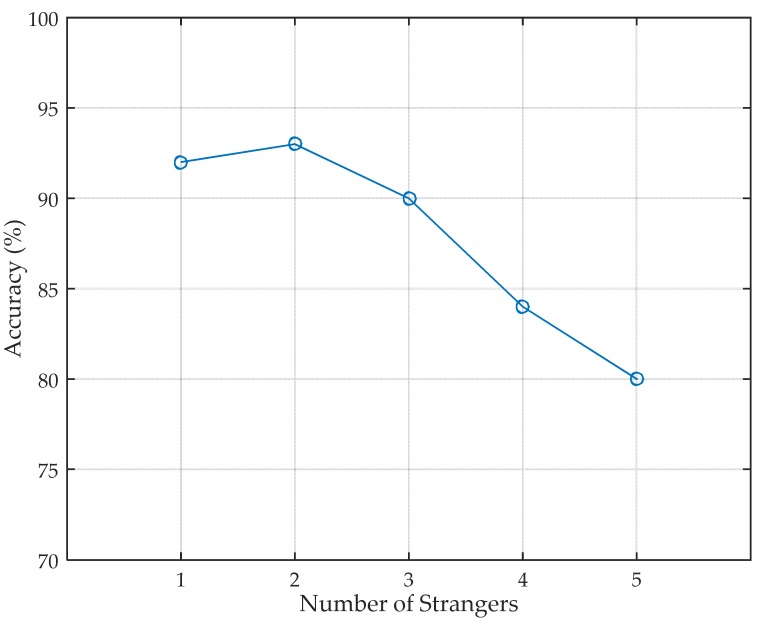
Performance of stranger recognition.

Stranger recognition is the first step of human identification in security system, so it is a crucial procedure of Wii. Before identifying the person’s identity, Wii has to recognize whether the person entering the monitoring area is an authenticated person or a stranger. After only five walking steps, Wii can determine whether the person is a stranger. When an authenticated person is recognized, the identity identification procedure is activated. The accuracy of stranger recognition under different number of strangers in the training set is shown in Figure 7. The strangers are randomly selected from the volunteers and modeled using the same features as the authenticated people. The selection is performed several times and the final result is the average accuracy of different combination. As can be seen, the overall trend of the accuracy decreases as the number of strangers in the training set becomes larger. This is because, as the number of strangers in the training set increases, the features of strangers become more and more generally representative that the features of authenticated people may be submerged in those of strangers. Consequently, the number of strangers in the training set should not be too large.

#### 5.2.2. Evaluation of Step Segmentation

**Figure 8:**
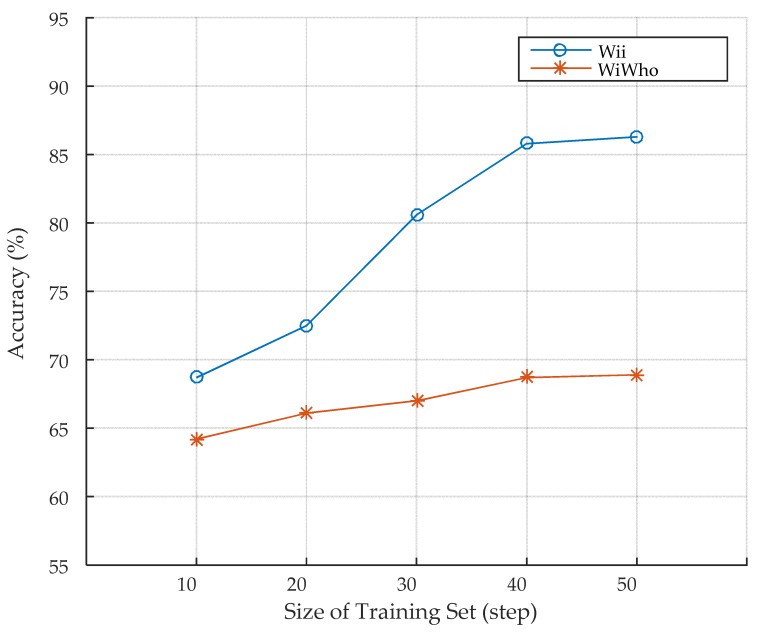
Step segmentation.

Step segmentation is a critical module of gait analysis in the system. To validate its effectiveness, we compare Wii with WiWho fairly using the same features and classifier as that of WiWho as well as the experimental setup. Concretely, the features include the feature ID of 1, 2, 5, 8, and 11 in Table 1 and the classifier is decision tree. There are eight volunteers in the training set. The size of the training set varies from 10 to 50 steps. The size of walking window is five steps. The identification accuracy of the two approaches is shown in Figure 8. It can be seen that the identification accuracy of both approaches rises as the size of training set grows. The accuracy of Wii grows faster and keeps higher than that of WiWho as the size of training set becomes larger. It indicates that the step segmentation module of Wii can extract more effective step information and has a better performance. 

#### 5.2.3. Identity Identification with Different Numbers of Features

**Figure 9:**
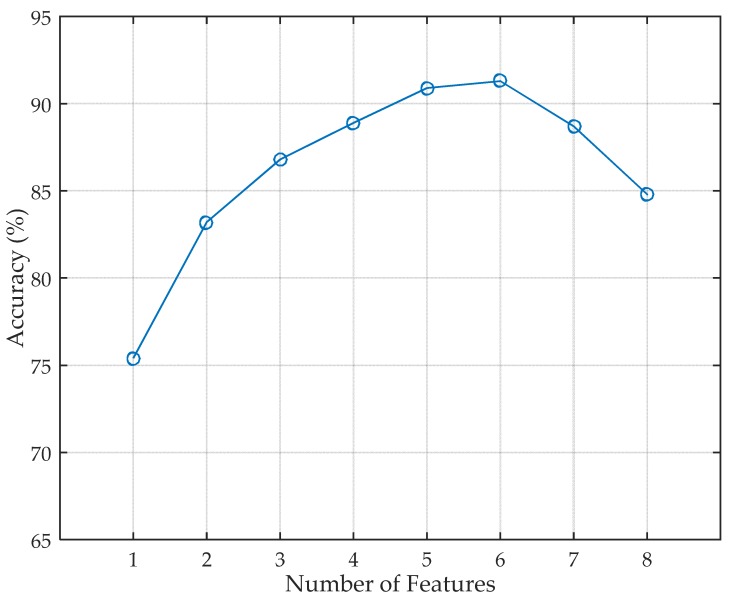
Impact of different numbers of features.

The number of features has impact on both the computation complexity and the identification accuracy. We select the first *n* features that have the highest information gain, and eight volunteers are included in this evaluation. The evaluation result is depicted in Figure 9. The identification accuracy first rises with the number of features. However, the accuracy is not changing monotonically with the number of features. It starts to decrease when the number of features becomes larger than 6 because an excess number of features leads to overfitting to the system. As a result, the five features with the highest information gain give a good trade-off between computation complexity and accuracy.

#### 5.2.4. Identity Identification with Different Sizes of Training Sets

**Figure 10:**
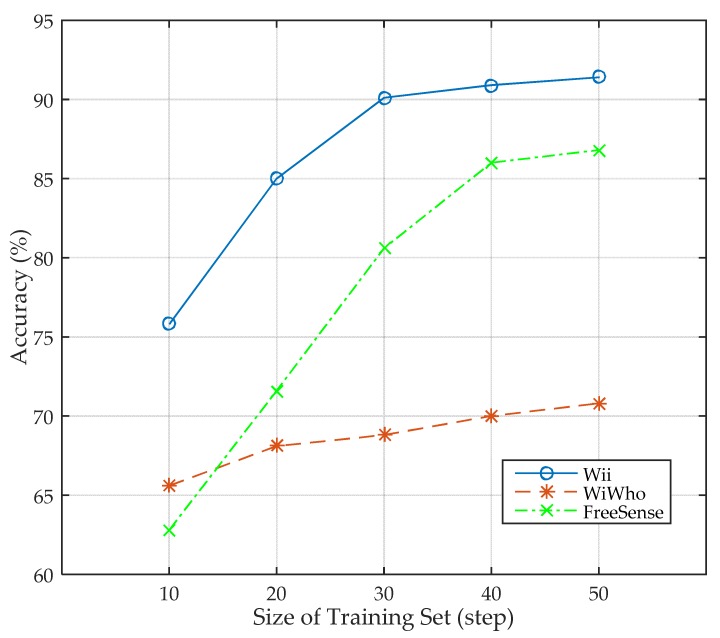
Impact of the size of training set.

**Figure 11:**
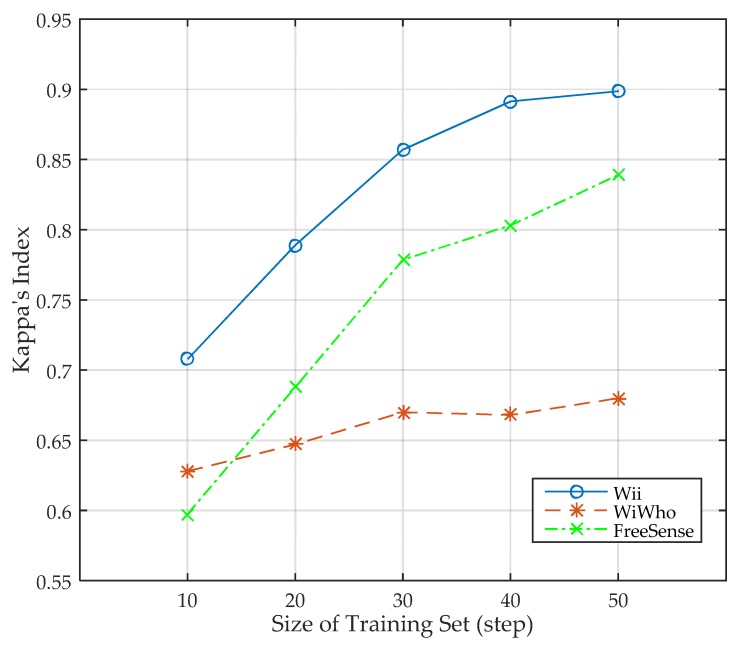
Kappa’s index under different sizes of training set.

In this section, we present the performance of Wii when the size of training set varies. The size of training set varies from 10 to 50 steps for one subject, and eight subjects are included in the training set. The size of the walking window is five steps. In addition, to study the advancement of Wii, we also compare the performance with WiWho and FreeSense. The design scenario of WiWho and FreeSense are almost the same as Wii that all of them work in home or small office environment. As a result, we implement WiWho and FreeSense using the same experimental setup as Wii. According to Figure 10, the accuracy of Wii keeps increasing from 75.8% to 91.4% as the size of training set grows. As indicated in the figure, the accuracy of Wii increases faster when the size of training set grows from 10 to 30, while slows down as it changes to 40 and 50. This means the performance of Wii becomes stable when the size is 40 or larger. The paired *t*-test is used to analyze the differences of the results between Wii and the other two methods. The test results are shown in Table 3, where P1 is the possibility of *t*-test between Wii and WiWho, while P2 is the possibility of *t*-test between Wii and FreeSense. As can be seen, all reject the null hypothesis, which means that the results are independent from sample selection. In addition, we use the Kappa’s index to evaluate the classification agreement [36]. The Kappa’s index of the three methods under different size of training set is shown in Figure 11. Wii has a higher Kappa’s index compared to the other two methods, which means that Wii has a higher agreement. In one word, Wii performs better than WiWho and FreeSense. It is because the features selected theoretically contain more information, and they are more suitable to the classifier and need a smaller training set.

#### 5.2.5. Identity Identification with Different Group Sizes

We then evaluate Wii with different group sizes. The size of the group increases from two to eight persons, and the size of training set is 40. We fix the size of training set to 40 steps because the performance rises to a stable level when the size of training set is larger than 30 according to Figure 10. For the cases that the size of the group is 2–7, we randomly select 2–7 persons from the eight volunteers, and Wii is trained before classification.

**Figure 12:**
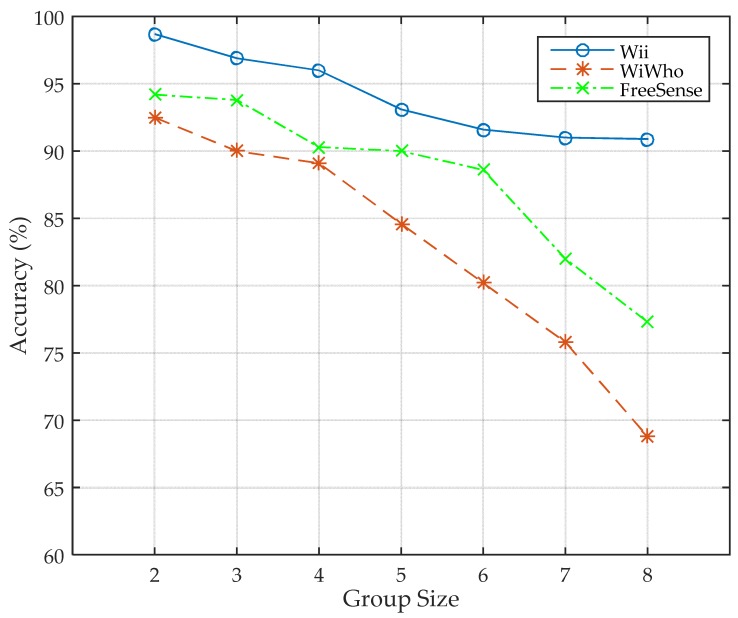
Performance with different group sizes.

As shown in Figure 12, the accuracy of Wii decreases from 98.7% to 90.9% as the group size gets larger. It is because there are similar features among different people, and when the group size gets larger, the similar features add more confusion into the system, which makes it more difficult to identify a person. The same trend also happens in WiWho and FreeSense, but the accuracy decreases quickly when the group size gets larger than 4. Consequently, the comparison result indicates that Wii performs more stably when the group size gets larger.

#### 5.2.6. Identification Accuracy of Different People

**Figure 13:**
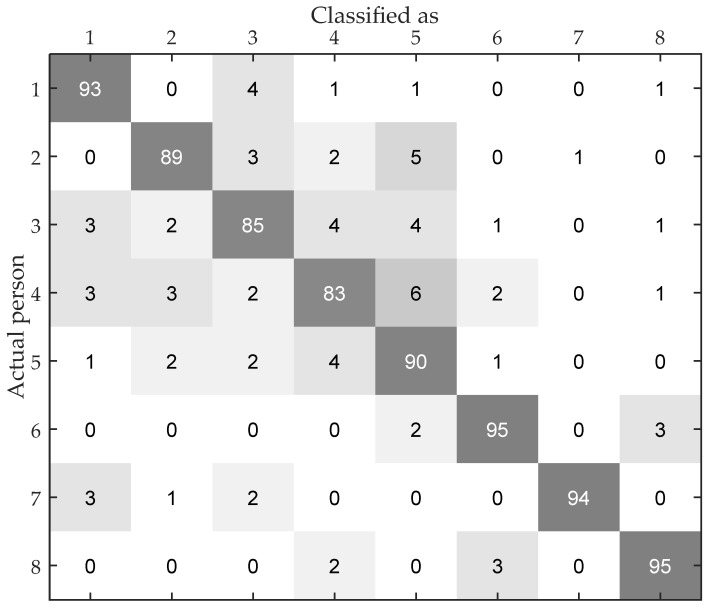
The confusion matrix of human identification with eight people.

The identification result of the eight volunteers in the above experiment is shown in the confusion matrix in Figure 13. It shows the details of the identification of different people. It is obvious that different people have different identification accuracy and it varies from 83% to 95%. According to Table 2, the result that Volunteer 6 and 8 have the highest accuracy as these two volunteers are the only female volunteers in the test group, which indicates that the gait pattern of female has less similarity with male. We can also find that, if two people have similar height and weight, they are more likely to have difficulties being correctly identified. Especially, the identification accuracy of Volunteer 7 is the highest among the male volunteers, as he has a higher height than the other male volunteers. In other words, height and weight have a significant impact on the transmission of wireless signal. 

#### 5.2.7. Impact of Walking Window Size

There is also another important factor in evaluation of Wii that can affect the identification accuracy. As FreeSense is not a window-based approach, it is excluded in the evaluation of different walking window sizes. We further compare the performance of Wii with WiWho under different walking window sizes when the size of the training set is 40 and the group size is 8. 

**Figure 14:**
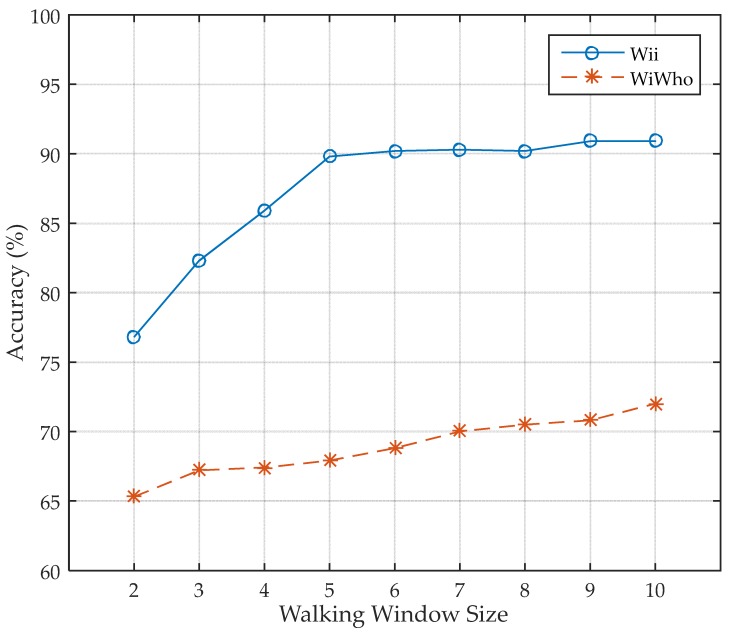
Impact of walking window size.

As can be seen in Figure 14, we change the size of walking window from 2 to 10. The smallest window size is 2 is because it is the smallest size larger than a single step. If the window size decreases to 1, the walking features decline to per-step features, which makes no sense. We think it is still practical for the system that the identification result comes out after the user walks for 10 steps if we can get a higher accuracy. From the evaluation result we can see that, when the size of the walking window is smaller than five steps, the identification accuracy of Wii keeps growing as the window size gets larger, which indicates that our walking features play a crucial role in improving the performance of identity identification. However, the accuracy becomes stable when the window size gets larger than five steps, which means that it is appropriate to set the size of walking window to 5. The accuracy of 90.9% can satisfy common smart home applications. Obviously, Wii can be better adopted in a larger group size.

## 6. Discussion

We did several evaluations in this work and showed the feasibility of identity identification using WiFi signals. However, there still exist some limitations in Wii. In this section, we will discuss the limitations and potentials of Wii, which give the direction of our future work.

Although Wii can achieve a relatively high identification accuracy, it can also be affected by many factors.

First, we have collected walking data in several locations and different paths. At first, we planned to identify people even if he changed his path. However, the extracted features change a lot when the person walks in another path. We cannot successfully identify a person when the training data and test data are collected at different paths. Specifically, we also find that the identification accuracy is higher when the person walks across the line-of-sight of the transceivers than other paths. As a result, we constraint the walking path in our experiments. 

In addition, people’s walking gait indeed has a great impact on the identification result. Thus, during data collection, the volunteers are asked to keep their walking gait and walk naturally. WLAN-based human identification has some similarities with radar techniques. Thus, different poses may cause different fingerprints for the same person, which influences the accuracy.

Furthermore, WiFi signals can be easily affected by environmental changes. Thus, Wii can only be used when there is only one person walking in the monitoring area without anyone else.

Despite these limitations, wireless signal-based identity identification technique has much potential. Besides intrusion detection, it can also be used as a critical function in behavior analysis systems as well as to provide personalized services in smart spaces. 

In our future work, we plan to explore new features that can represent people’s gait patterns more accurately in order to make the identity identification system have the ability to be adaptively used even when the user walks in different paths.

## 7. Conclusions

In this paper, we propose an effective identity identification approach Wii only based on WiFi signals which brings much convenience in smart home applications. It utilizes existing WLAN infrastructures and is based on fine-grained CSI from physical layer of wireless network. Wii divides time series of walking into segments by steps and extracts both time and frequency domain features from per-step and walking segment perspective. The extracted feature can be properly used in representing human’s gait patterns. It can effectively recognize strangers. To evaluate the performance of Wii, we implement a set of experiments from several perspectives. The results show that Wii achieves an average identification accuracy of 98.7% when there are two people, and 90.9% when there are eight people, which is effective and can satisfy typical home security. The limitations and potentials of WiFi signal-based identity identification systems are also discussed in this work.

## Figures and Tables

**Table 1:** Candidate features.

ID	Feature Name	Description
1	Maximum (max)	$max=\max(cpl^{s})$ (6)
2	Minimum (min)	$min=\min(cpl^{s})$ (7)
3	Mean	$mean=\frac{1}{n}\sum_{i=1}^{n}cpl^{s}{}_{i}$ (8)
4	Median	$median=\mathrm{median}(cpl^{s})$ (9)
5	Standard deviation (std)	$std=\sqrt{\frac{1}{n}\sum_{i=1}^{n}(cpl_{i}^{s}-mean{)}^{2}}$ (10)
6	Skewness (sk)	$sk=\frac{1}{n}\sum_{i=1}^{n}{\left(cpl_{i}^{s}-mean\right)}^{2}$ (11)
7	Kurtosis (ku)	$ku=\frac{\sum_{i=1}^{n}{\left(cpl_{i}^{s}-mean\right)}^{4}}{(n-1)std^{4}}$ (12)
8	Average step time (ast)	$ast=\frac{1}{m}\sum_{j=1}^{m}\mathrm{len}(cpl^{s}{}_{j})$ (13)
9	Variance of step time (vst)	$vst=\frac{1}{m}\sum_{j=1}^{m}{\left(\mathrm{len}(cpl^{s}{}_{j})-ast\right)}^{2}$ (14)
10	Energy	$energy=\sum_{i=1}^{n}{\left(cpl_{i}^{s}\right)}^{2}$ (15)
11	Entropy	$entropy=-\sum_{k=1}^{10}p_{k}\log p_{k}$ (16)

**Table 2:** Basic information of volunteers.

Volunteers	Gender	Height (cm)	Weight (kg)	Age
1	male	174	63	29
2	male	175	70	26
3	male	175	66	27
4	male	170	62	25
5	male	171	60	25
6	female	164	50	24
7	male	183	74	24
8	female	161	50	24

**Table 3:** P values of the paired *t*-test of different sizes of training set.

Size of Training Set (Step)	10	20	30	40	50
P1	0.007	0.007	0.005	0.005	0.005
P2	0.007	0.010	0.016	0.014	0.018
